# Spectral-like conjugate gradient methods with sufficient descent property for vector optimization

**DOI:** 10.1371/journal.pone.0302441

**Published:** 2024-05-15

**Authors:** Jamilu Yahaya, Poom Kumam, Sani Salisu, Kanokwan Sitthithakerngkiet

**Affiliations:** 1 Center of Excellence in Theoretical and Computational Science (TaCS-CoE) and KMUTTFixed Point, Research Laboratory, Room SCL 802 Fixed Point Laboratory Science Laboratory Building, Department of Mathematics, Faculty of Science, King Mongkut’s University of Technology Thonburi (KMUTT), Thung Khru, Bangkok, Thailand; 2 NCAO Research Center, Fixed Point Theory and Applications Research Group, Center of Excellence in Theoreticaland Computational Science (TaCSCoE), Faculty of Science, King Mongkut’s University of Technology Thonburi (KMUTT), Thung Khru, Bangkok, Thailand; 3 Department of Mathematics, Faculty of Physical Sciences, Ahmadu Bello University Zaria, Kaduna, Nigeria; 4 Department of Mathematics, Faculty of Natural and Applied Sciences, Sule Lamido University Kafin Hausa, Jigawa, Nigeria; 5 Intelligent and Nonlinear Dynamic Innovations Research Center, Department of Mathematics, Faculty of Applied Science, King Mongkut’s University of Technology North Bangkok (KMUTNB), Bangsue, Bangkok, Thailand; Northwestern Polytechnical University, CHINA

## Abstract

Several conjugate gradient (CG) parameters resulted in promising methods for optimization problems. However, it turns out that some of these parameters, for example, ‘PRP,’ ‘HS,’ and ‘DL,’ do not guarantee sufficient descent of the search direction. In this work, we introduce new spectral-like CG methods that achieve sufficient descent property independently of any line search (LSE) and for arbitrary nonnegative CG parameters. We establish the global convergence of these methods for four different parameters using Wolfe LSE. Our algorithm achieves this without regular restart and assumption of convexity regarding the objective functions. The sequences generated by our algorithm identify points that satisfy the first-order necessary condition for Pareto optimality. We conduct computational experiments to showcase the implementation and effectiveness of the proposed methods. The proposed spectral-like methods, namely nonnegative SPRP, SHZ, SDL, and SHS, exhibit superior performance based on their arrangement, outperforming HZ and SP methods in terms of the number of iterations, function evaluations, and gradient evaluations.

## 1 Introduction

In recent times, the successful application of CG methods in solving vector optimization problems (VOPs) has attracted considerable attention as detailed in [1]. Since then, these approaches have gained recognition for their simplicity and minimal memory requirements, thereby proving effective (see, for example, [2, 3] and their references).

Before exploring VOPs, let us consider some well-known CG parameters related to the natural unconstrained optimization problem, which focuses on minimizing $\bar{f}:R^{n}\to R.$ The parameters include the *β*_*k*_ of Polak-Ribiére–Polyak (PRP) [4], Hestenes-Stiefel (HS) [5], Dai–Liao (DL) [6], and Hager–Zhang (HZ) [7, 8]. Other well-known CG methods include: a survey on DL [9], Fletcher-Reeves (FR) [10], Conjugate Descent (CD) [11], Dai-Yuan (DY) [12], and Liu-Storey (LS) [13]. In most cases, the convergence of the CG method based on these parameters is achieved only if the search direction attains a decent property or sufficient descent condition.

Another crucial iterative technique for optimization is the spectral gradient method introduced in [14]. This method has substantial performance. Later, in [15] the spectral gradient and CG method were combined to give the first spectral conjugate gradient (SCG) method. The method used the following search direction

$$\begin{array}{c} d_{k}≔-g_{k},\;\;\;k=0,\;\;\;d_{k}≔-\theta_{k}g_{k}+\beta_{k}s_{k-1},\;\;\;k\geq 1 \end{array}$$ (1)

with spectral parameter *θ*_*k*−1_ and *β*_*k*_, see [15]. The SCG method has been extensively investigated by several authors, including spectral CG with sufficient descent property [16, 17], spectral CG involving RMIL [18], and self-adjusting spectral involving hybrid DL CG [19].

Most of the parameters mentioned above are considered for VOP, in which the objective function *F* represents a function that maps vectors from an *n*-dimensional space of real numbers to vectors in an *m*-dimensional space of real numbers. *F* is also assumed to be a continuously differentiable function. Let *Q* be a subset of the *m*-dimensional space of real numbers, closed, convex, and a pointed cone with a nonempty interior. The unconstrained VOP is defined as

$$\begin{array}{c} \text{VOP:}\;\;\;\;\;\;{\text{Opt}}_{Q}\;\;F\left(t\right),\;\;\;\;\;\; \end{array}$$ (2)

which optimizes *F*, where $t\in R^{n}.$ Note that, *F* = (*F*_1_, ⋯, *F*_*m*_)^*T*^ for some $F_{i}:R^{n}\to R,$ *i* = 1, ⋯, *m*. Moreover, if *Q* is the *m*-dimensional space of real numbers with nonnegative components, then VOP reduces to multi-objective optimization (MOO). Also, if *Q* consists of only nonnegative real numbers and *m* = 1, then (2) reduces to single-objective optimization (SOO).

Several applications in industry and finance are considered instances of VOP, where multiple objective functions are optimized concurrently. Consequently, it becomes imperative to determine a set of optimal points for VOP [20–26]. Because a total order is lacking in $R^{m}$ where *m* ≥ 2, the solution to VOP consists of a set of non-dominated points, often referred to as Pareto optimal or efficient points. The challenge lies in identifying the solutions that strike the most advantageous balance. It is important to mention that, (2) signifies minimizing *F* with respect to the ordering cone *Q*.

One way to approach VOPs is the scalarization techniques, which parameterized single-objective optimization problems to yield Pareto-optimal points. The decision-maker must choose the parameters, as they are not predetermined. Making this choice can pose significant challenges or become impossible for some problems [27–29]. Consequently, to overcome these drawbacks, some descent-based algorithms have been suggested as solution approaches for VOP. Thanks to the works in [30, 31]. Subsequently, numerous other studies have followed this trajectory, exploring similar directions, see the survey on MOO descent methods in [32] and the references [33–36].

In [1], the conjugate parameters of [4, 5, 10, 12, 37] are considered for VOPs. Their study encompassed numerical implementations of these methods, which were analyzed and discussed. Among these methods, as per the considered test problems, the nonnegative PRP and HS showed exceptional performance in comparison to the others. Conversely, the CD and DY methods outperformed FR in terms of efficiency. Thereafter, Goncalves and Prudente [38] extended the Hager-Zhang CG method for VOPs. For this method, the search direction does not guarantee the descent condition, even with an exact LSE. To address this issue, the authors proposed a self-adjusting HZ method, utilizing a sufficiently accurate LSE, which possesses the descent property. Other works in this direction include the work of [39] based on sufficiently accurate LSE, the first hybrid CG methods proposed for VOPs in [3] and some modified CG methods in [40].

Following the works in [15, 41, 42], He et al. [43] proposed the SCG methods for VOPs. In contrast to scalar optimization, the extension of SCG does not yield a descent property. As a result, the authors provided a modified self-adjusting SCG algorithm to induce the property through the algorithm. They established the convergence using a sufficiently accurate LSE that satisfies the Wolfe LSE. It is therefore natural to ask if there could be an SCG method for which the descent property is guaranteed without inducing it into the algorithm.

In this paper, we affirmatively address the above question. We define a new form of search direction in the vector context, inspired by the work of [44]. Our method yields a sufficient descent property independent of any LSE for arbitrary nonnegative conjugate parameters. We consider four of these parameters and establish their convergence using Wolfe LSE. We provide computational experiments to validate our findings. The outcomes here are compared with HZ and SP methods, showing that our proposed methods are promising.

The presentation of the work proceeds as follows: Section 2 discusses the basic notions and preliminaries. Section 3 presents the proposed algorithm and its convergence properties. Section 4 presents and discusses computational experiments, and Section 5 provides substantial remarks.

## 2 Preliminaries

This section presents the basic notions related to VOPs. For further details, see [1, 31, 45–47]. Throughout the subsequent sections, (2) signifies minimizing *F* with respect to the ordering cone *Q*. Moreover, for a generic *Q*, the partial order in $R^{m}$ generated by *Q*, ≼_*Q*_ is in the sense that *z* ≼_*Q*_ *y* ⇔ *y* − *z* ∈ *Q*, and ≺_*Q*_, is given by *z* ≺_*Q*_ *y* ⇔ *y* − *z* ∈ *int*(*Q*). Moreover, the idea of optimality is substituted with “Pareto-optimal or efficient” and “weak Pareto-optimal or efficient” in VOP.

**Definition 1** [32] *A vector* $\bar{t}\in R^{n}$ *is Pareto-optimal (efficient) if and only if* ∄ *a vector* $y\in R^{n}$ *s.t* $F\left(y\right){≼}_{Q}F\left(\bar{t}\right)$ *and* $F\left(y\right)\neq F\left(\bar{t}\right)$.

**Definition 2** [32] *A vector* $\bar{t}\in R^{n}$ *is weak Pareto-optimal (weak efficient) if and only if* ∄ *a vector* $y\in R^{n}$ *s.t* $F\left(y\right){≺}_{Q}F\left(\bar{t}\right)$.

**Remark 3** *Definition 1 implies Definition 2, but the converse is not generally true*.

Below are some properties of the cone *Q*, including its positive polar cone:

$$\begin{array}{c} Q^{*}≔\left\{q\in R^{m}\;|\;\left\langle q,t\right\rangle \geq 0,\;\forall \;t\in Q\right\} \end{array}$$

Notice that *Q* = Q**, considering the convexity and closedness of *Q*,

$$\begin{array}{c} -Q=\left\{t\in R^{m}\;|\;\left\langle t,q\right\rangle \leq 0,\;\forall \;q\in Q^{*}\right\}\;and\;-int\left(Q\right)=\left\{t\in R^{m}\;|\;\left\langle t,q\right\rangle <0,\;\forall \;q\in Q^{*}\backslash \left\{0\right\}\right\}. \end{array}$$

The cone formed by $W\subseteq R^{m}$ is denoted as *cone*(*W*), and *conv*(*W*) represents the convex hull of *W*. Now, consider *K* ⊆ Q*, where 0 ∉ *K* and *K* is compact. We define Q* as follows:

$$\begin{array}{c} Q^{*}=cone\left(conv\left(K\right)\right). \end{array}$$ (3)

Now, for a generic set *Q*, we defined *K* as follows:

$$\begin{array}{c} K=\{q\in Q^{*}\;|\;||q||=1\}, \end{array}$$ (4)

which satisfies the condition in (3). In this work, we adopt the definition of *K* provided in (4). The Jacobian of *F* at *t* is denoted as *JF*(*t*) and the image of *JF*(*t*) on $R^{m}$ is represented as Image(*JF*(*t*)).

The following

$$\begin{array}{c} -int\left(Q\right)\cap Image(JF\left(\bar{t}\right))=\emptyset , \end{array}$$ (5)

is considered the necessary condition for *Q* − *optimality* of $\bar{t}\in R^{n}.$ If condition (5) is satisfied, the point $\bar{t}\in R^{n}$ is termed as *stationary or Q-critical*. Conversely, if $\bar{t}\in R^{n}$ is not *Q*-critical, there exists a vector $b\in R^{n}$ s.t $JF\left(\bar{t}\right)b\in -\text{int}\left(Q\right)$, indicating that *b* is a *Q*-descent direction (*Q*-DD) at $\bar{t}$. See, for example [47].

Define $\zeta :R^{m}\to R$ as

$$\begin{array}{c} \zeta (t)≔\text{sup}\{\langle t,q\rangle \;\;|\;\;q\in K\}. \end{array}$$

The *ζ* is well-defined, since *K* is compact. We observed that *ζ* also provides some features of −*Q* and −*int*(*Q*) as follows: $-Q=\{t\in R^{m}\;|\;\zeta \left(t\right)\leq 0\}$ and $-int\left(Q\right)=\{t\in R^{m}\;|\;\zeta \left(t\right)<0\}$.

Next, let us define $f:R^{n}\times R^{n}\to R$ by

$$\begin{array}{c} f(t,d)≔\zeta (JF(t)d)=\text{sup}\{\langle JF(t)d,\;q\rangle \;|\;q\in K\}. \end{array}$$ (6)

**Definition 4** *We have a Q-DD if f*(*t*, *d*) < 0, *and a Q-critical point if f*(*t*, *d*) ≥ 0 *for all d*.

**Definition 5** *If* $d\in R^{n}$ *satisfies*

$$\begin{array}{c} f(t,d)\leq cf(t,h(t)), \end{array}$$ (7)

*then we have a sufficient descent condition (SDC), where c* > 0.

**Lemma 6** [31]. *If* $F:R^{n}\to R^{m}$ *is in C*^1^. *Then, the following statements hold*:

*(a)* $f\left(t,t^{\prime }+\ell d\right)\leq f\left(t,t^{\prime }\right)+\ell f\left(t,d\right)$ , *for* $t,t^{\prime },d\in R^{n}$ *and ℓ* ≥ 0;

*(b) The mapping* (*t*, *d*) ↦ *f*(*t*, *d*) *is continuous*;

*(c)* |*f*(*t*, *d*) − *f*(*t*′, *d*)| ≤ ‖*JF*(*t*) − *JF*(*t*′)‖|‖*d*‖, $\forall \;\;\;t,t^{\prime },d\in R^{n}$;

*(d) If* ‖*JF*(*t*) − *JF*(*t*′)‖ ≤ *L*‖*t* − *t*′‖, *then* ‖*f*(*t*, *d*) − *f*(*t*′, *d*)‖ ≤ *L*‖*d*‖‖*t* − *t*′‖.

For CG method of VOPs, we define $h:R^{n}\to R^{n}$ by

$$\begin{array}{c} h\left(t\right)≔\text{argmin}\{f\left(t,d\right)+\frac{{\left\|d\right\|}^{2}}{2}\;|\;d\in R^{n}\}, \end{array}$$ (8)

and $v:R^{n}\to R$ by

$$\begin{array}{c} v\left(t\right)≔f\left(t,h\left(t\right)\right)+\frac{{\left\|h\left(t\right)\right\|}^{2}}{2}. \end{array}$$ (9)

The iterate begins with arbitrary $t_{0}\in R^{n}$ and updated

$$\begin{array}{c} t_{k+1}=t_{k}+\ell_{k}d_{k},\;\;\;\;\;\;k\geq 1, \end{array}$$ (10)

where *ℓ*_*k*_ > 0 is called the step size, computed via an LSE method, and the search direction *d*_*k*_ is defined as

$$\begin{array}{c} d_{k}≔\{\begin{array}{c} h(t_{k}),\;\;\;\;\;\;\;\;k=0,\\ h\left(t_{k}\right)+\beta_{k}d_{k-1},\;\;\;k\geq 1, \end{array} \end{array}$$ (11)

with *β*_*k*_ as the conjugate parameter. The VOPs versions of parameters proposed in [1] are as follows:

$$\begin{array}{c} \beta_{k}^{FR}≔\frac{f(t_{k},h\left(t_{k}\right))}{f(t_{k-1},h\left(t_{k-1}\right))},\;\beta_{k}^{CD}≔\frac{f(t_{k},h\left(t_{k}\right))}{f(t_{k-1},d_{k-1})},\;\beta_{k}^{DY}≔\frac{-f(t_{k},h\left(t_{k}\right))}{f\left(t_{k},d_{k-1}\right)-f\left(t_{k-1},d_{k-1}\right)}, \end{array}$$

$$\begin{array}{c} \beta_{k}^{PRP}≔\frac{-f\left(t_{k},h\left(t_{k}\right)\right)+f\left(t_{k-1},h\left(t_{k}\right)\right)}{-f(t_{k-1},h\left(t_{k-1}\right))},\;\beta_{k}^{HS}≔\frac{-f\left(t_{k},h\left(t_{k}\right)\right)+f\left(t_{k-1},h\left(t_{k}\right)\right)}{f\left(t_{k},d_{k-1}\right)-f\left(t_{k-1},d_{k-1}\right)}, \end{array}$$ (12)

where *f*(⋅, ⋅) is defined by Eq (6). The vector version of Hager-Zhang was given in [38] as follows:

$$\begin{array}{c} \beta_{k}^{HZ}≔\frac{1}{\omega_{2}-\omega_{4}}(\omega_{1}-\mu \omega_{2}\frac{\omega_{1}+\omega_{3}}{\omega_{2}-\omega_{4}}), \end{array}$$ (13)

where $\mu >\frac{1}{4}$ and *ω*_1_ ≔ −*f*(*t*_*k*_, *h*(*t*_*k*_)) + *f*(*t*_*k*−1_, *h*(*t*_*k*_)), *ω*_2_ ≔ *f*(*t*_*k*_, *d*_*k*−1_), *ω*_3_ ≔ *f*(*t*_*k*_, *h*(*t*_*k*−1_)) − *f*(*t*_*k*−1_, *h*(*t*_*k*−1_)), *ω*_4_ ≔ *f*(*t*_*k*−1_, *d*_*k*−1_), with

$$\begin{array}{c} \beta_{k}≔\text{max}\left\{\beta_{k}^{HZ},\eta_{k}\right\}, \end{array}$$ (14)

where

$$\begin{array}{c} \eta_{k}≔\frac{-1}{\|d_{k}\|\text{min}\{\eta ,\|h\left(t_{k}\right)\left\|\right\}},\;\;\;\eta >0. \end{array}$$

Now, consider the problem:

$$\begin{array}{c} \begin{array}{cc} \text{Minimize}\; & \;\ell +\frac{1}{2}{\left\|d\right\|}^{2},\\ \text{s.t}\; & \;{\left[JF\left(t\right)d\right]}_{i}\leq \ell ,\;\;i=1,2,\cdots ,m, \end{array} \end{array}$$ (15)

see for instance, [46].

Here, we define the most commonly known LSE used for conjugate gradient algorithms, namely the exact and inexact LSE, which are defined as follows: we have an exact LSE if *ℓ* > 0 is computed as follows:

$$\begin{array}{c} f(t+\ell d,d)=0. \end{array}$$ (16)

As stated in [1], the standard Wolfe LSE and the strong Wolfe LSE for VOPs. It state that *ℓ* > 0 fulfills the *standard Wolfe condition* (WWC) if

$$\begin{array}{c} \begin{array}{c} F\left(t+\ell d\right){≼}_{Q}F\left(t\right)+\rho \ell f\left(t,d\right)e\\ f(t+\ell d,d)\geq \sigma f(t,d). \end{array} \end{array}$$ (17)

We find *ℓ* > 0 by means of the *strong Wolfe condition* (SWC) if the following conditions are satisfied:

$$\begin{array}{c} \begin{array}{c} F\left(t+\ell d\right){≼}_{Q}F\left(t\right)+\rho \ell f\left(t,d\right)e\\ |f(t+\ell d,d)|\leq \sigma |f(t,d)|, \end{array} \end{array}$$ (18)

where *e* ∈ *Q* s.t

$$\begin{array}{c} 0<\langle q,\;e\rangle \leq 1, \end{array}$$ (19)

and 0 < *ρ* < *σ* < 1.

**Lemma 7** [31] *Consider h*(*t*) *and v*(*t*) *as defined in* (8) *and* (9) *respectively, then we have*

(a) *if t is a Q-critical point, then h*(*t*) = 0 *and v*(*t*) = 0,
(b) *let t be not Q-critical point, then h*(*t*) ≠ 0, *v*(*t*) < 0, $f\left(t,h\left(t\right)\right)<-\frac{{\left\|h\left(t\right)\right\|}^{2}}{2}<0$ *and h*(*t*) *is a Q-DD*,
(c) *the maps*, *h and v are continuous*.

## 3 Spectral-like algorithm and convergence properties

In this section, we present the main algorithm and its convergence properties. However, before delving into details, it is important to consider the following throughout this work: according to Lemma 7 (b), we have *h*(*t*_*k*_) ≠ 0. Otherwise, *t*_*k*_ is a *Q*-critical or stationary point.

Consider the vector version of the Dai–Liao conjugate parameter as follows:

$$\begin{array}{c} \beta_{k}^{DL}≔\frac{-f\left(t_{k},h\left(t_{k}\right)\right)+f\left(t_{k-1},h\left(t_{k}\right)\right)}{f\left(t_{k},d_{k-1}\right)-f\left(t_{k-1},d_{k-1}\right)}-\alpha \frac{f(t_{k},s_{k-1})}{f\left(t_{k},d_{k-1}\right)-f\left(t_{k-1},d_{k-1}\right)}, \end{array}$$ (20)

where *α* > 0. Now, we consider the following nonnegative parameters

$$\begin{array}{c} \begin{array}{cc} & \beta_{k}=\beta_{k}^{PRP+}=\text{max}\left\{\beta_{k}^{PRP},0\right\};\\ & \beta_{k}=\beta_{k}^{HS+}=\text{max}\left\{\beta_{k}^{HS},0\right\};\\ & \beta_{k}=\beta_{k}^{HZ+}=\text{max}\left\{\beta_{k}^{HZ},0\right\};\\ & \beta_{k}=\beta_{k}^{DL+}=\text{max}\left\{\beta_{k}^{DL},0\right\}, \end{array} \end{array}$$ (21)

and the iterative:

$$\begin{array}{c} t_{k+1}=t_{k}+\ell_{k}d_{k},\;\;\;\;\;\;k\geq 1, \end{array}$$ (22)

where *d*_*k*_ is defined as follows:

$$\begin{array}{c} d_{k}≔\{\begin{array}{cc} h(t_{k}), & k=0\;\;or\;\;f(t_{k},s_{k-1})<0\\ h\left(t_{k}\right)+\beta_{k}s_{k-1}+\beta_{k}\frac{f(t_{k},s_{k-1})}{-f(t_{k},h\left(t_{k}\right))}h\left(t_{k}\right), & f(t_{k},s_{k-1})\geq 0,\;\;and\;k\geq 1. \end{array} \end{array}$$ (23)

As mentioned in the preliminary section, *ℓ*_*k*_ > 0 is computed via an LSE strategy, *s*_*k*−1_ = *t*_*k*_ − *t*_*k*−1_ = *ℓ*_*k*−1_*d*_*k*−1_, and *β*_*k*_ is a nonnegative parameter. Observe that by Lemma 7 (b), we have *f*(*t*_*k*_, *h*(*t*_*k*_)) ≠ 0.

The following sufficient descent condition follows from Lemma 6(a) and (23)

$$\begin{array}{c} f\left(t_{k},d_{k}\right)\leq f\left(t_{k},h\left(t_{k}\right)\right),\;\;\forall \;\;k\geq 1. \end{array}$$ (24)

This implies that we always have (7) with *c* ≤ 1, irrespective of LSE and the *β*_*k*_ parameter.

**Remark 8** *It is easy to see that* (23) *can be expressed as*

$$\begin{array}{c} d_{k}={\bar{\theta }}_{k}h\left(t_{k}\right)+\beta_{k}s_{k-1}, \end{array}$$ (25)

*where* ${\bar{\theta }}_{k}=1+\beta_{k}\frac{f(t_{k},s_{k-1})}{-f(t_{k},h\left(t_{k}\right))}.$ *This implies that our method is a special case of SCG method with* ${\bar{\theta }}_{k}=1+\beta_{k}\frac{f(t_{k},s_{k-1})}{-f(t_{k},h\left(t_{k}\right))}$. *Thus, we now have a spectral CG that achieved* (7) *without any LSE. Note that when employing an exact LSE here*, (23) *becomes the well-known nonlinear CG method* (11) *with d*_*k*−1_ *replaced by s*_*k*−1_.

Before we proceed to the convergence analysis, we will require the following significant assumptions.

**Assumption 9** *Let Q be composed of a finite number of elements and* ∃ *an open set* Ω *s.t* $L≔\{t\;\;|\;\;F\left(t\right){≼}_{Q}F\left(t_{0}\right)\}\subset \Omega ,$ *where* $t_{0}\in R^{n}$ *and* ∃ *L* > 0 *s.t* ‖*JF*(*t*) − *JF*(*t*′)‖ ≤ *L*‖*t* − *t*′‖ *for all t*, *t*′ ∈ Ω.

**Assumption 10** *Let* ${\left\{D_{k}\right\}}_{k\in N}\subset F\left(L\right)$ *and* *D*_*k*+1_ ≼_*Q*_ *D*_*k*_, *for all k, then* ∃ $D\in R^{m}$ *s.t* $D{≼}_{Q}D_{k}$.

**Assumption 11** *The set* $L≔\{t\;\;|\;\;F\left(t\right){≼}_{Q}F\left(t_{0}\right)\}$ *is bounded*.

Note that, by Assumption 11 we have that $\{t_{k}\}\subset L,$ this implies that, ∃ $\bar{M}>0$ s.t

$$\begin{array}{c} \|t_{k}\|\leq \bar{M}, \end{array}$$ (26)

for all *k*. Therefore, we have from Lemma 6(d) that ∃ *γ* > 0 s.t

$$\begin{array}{c} \|JF(t_{k})\|\leq \gamma , \end{array}$$ (27)

for all *k*. Also, by the boundedness of {*f*(*t*_*k*_, *h*(*t*_*k*_))} and Lemma 7(b), there exists *δ* > 0 s.t

$$\begin{array}{c} \|h(t_{k})\|\leq \delta , \end{array}$$ (28)

for all *k*. By (27) and (28), we have

$$\begin{array}{c} 0<-f\left(t_{k},h\left(t_{k}\right)\right)\leq \left\langle JF\left(t_{k}\right)h\left(t_{k}\right),q\right\rangle \leq \|JF\left(t_{k}\right)\|\|h\left(t_{k}\right)\|\leq \delta \gamma , \end{array}$$ (29)

with ‖*q*‖ = 1. We stress that these assumptions naturally extend those considered in single-objective optimization.

We will need the following Zoutendijk Lemma in our convergence analysis.

**Lemma 12** [1] *Let Assumptions 9 and 10 hold. Consider* (10), *with Q-DD d*_*k*_ *and ℓ*_*k*_ *satisfies* (17). *Then*,

$$\begin{array}{c} \sum_{k=0}^{\infty }\frac{f^{2}\left(t_{k},d_{k}\right)}{\left|\right|d_{k}{\left|\right|}^{2}}<+\infty . \end{array}$$ (30)

Next, we present the spectral-like algorithm for the nonnegative CG methods for VOPs.

**Algorithm 1**: A spectral-like algorithm for VOPs

**Step 0**: Take $t_{0}\in R^{n}$ and initialize *k* ← 1.

**Step 1**: Compute *h*(*t*_*k*_) and *v*(*t*_*k*_) as in (8) and (9), respectively.

**Step 2**: If *v*(*t*_*k*_) = 0, then stop. Otherwise, compute *ℓ*_*k*_ > 0 s.t condition (18) is satisfies.

**Step 3**: Compute *d*_*k*_ as defined in (23), where *β*_*k*_ is a nonnegative parameter.

**Step 4**: Set *t*_*k*+1_ = *t*_*k*_ + *ℓ*_*k*_*d*_*k*_, for *k* ← *k* + 1 and move to **Step 1**.

**Remark 13** *(i) Step 1 is well explained by Lemma 7*.

*(ii) In Step 2, we use a LSE procedure to compute ℓ*_*k*_ > 0 *fulfilling* (18). *We emphasize there exist ℓ*_*k*_ > 0 *fulfilling* (18) *under Assumptions 9 and 11 as detailed in* [1, 48].

*(iii) In Step 3, we compute d_k_ and utilize any of the conjugate parameters in* (21) *one at a time and move to the next step, where the iterates are updated continuously*.

One of the sufficient conditions for establishing the convergence here is to estimate the norm of its search direction as follows: if *k* = 0 or *f*(*t*_*k*_, *s*_*k*−1_) < 0, then we have the following estimate

$$\begin{array}{c} \|d_{k}\|=\|h\left(t_{k}\right)\|. \end{array}$$

Otherwise, we have from (23) that

$$\begin{array}{c} \|d_{k}{\|}^{2}={\left\|h\left(t_{k}\right)+\beta_{k}(s_{k-1}-\frac{f(t_{k},s_{k-1})}{f(t_{k},h\left(t_{k}\right))}h\left(t_{k}\right))\right\|}^{2} \end{array}$$

$$\begin{array}{c} \;\;\;\leq 2\|h\left(t_{k}\right){\|}^{2}+2{\left(\beta_{k}\right)}^{2}{\left\|s_{k-1}-\frac{f(t_{k},s_{k-1})}{f(t_{k},h\left(t_{k}\right))}h\left(t_{k}\right)\right\|}^{2}. \end{array}$$ (31)

Moreover, we get

$$\begin{array}{c} \|s_{k-1}-\frac{f(t_{k},s_{k-1})}{f(t_{k},h\left(t_{k}\right))}h\left(t_{k}\right){\|}^{2}\leq 2(\|s_{k-1}{\|}^{2}+\frac{f^{2}\left(t_{k},s_{k-1}\right)}{f^{2}\left(t_{k},h\left(t_{k}\right)\right)}{\left\|h\left(t_{k}\right)\right\|}^{2}) \end{array}$$

$$\begin{array}{c} \;\;\;\;=2(\frac{\|s_{k-1}{\|}^{2}f^{2}\left(t_{k},h\left(t_{k}\right)\right)+f^{2}\left(t_{k},s_{k-1}\right){\left\|h\left(t_{k}\right)\right\|}^{2}}{f^{2}\left(t_{k},h\left(t_{k}\right)\right)}). \end{array}$$ (32)

Note that, by (27) and (28), we have

$$\begin{array}{c} |f\left(t_{k},s_{k-1}\right){|}^{2}\leq \|JF\left(t_{k}\right){\|}^{2}\|s_{k-1}{\|}^{2}\leq \gamma^{2}{\left\|s_{k-1}\right\|}^{2},\;\;for\;all\;\;k, \end{array}$$ (33)

and

$$\begin{array}{c} |f\left(t_{k},h\left(t_{k}\right)\right){|}^{2}\leq \|JF\left(t_{k}\right){\|}^{2}{\left\|h\left(t_{k}\right)\right\|}^{2}\leq \gamma^{2}\delta^{2},\;\;for\;all\;\;k. \end{array}$$ (34)

Now, applying (33) and (34) in (32), we have

$$\begin{array}{c} \|s_{k-1}-\frac{f(t_{k},s_{k-1})}{f(t_{k},h\left(t_{k}\right))}h\left(t_{k}\right){\|}^{2}\leq (\frac{2\gamma \delta \|s_{k-1}\|}{\left|f(t_{k},h\left(t_{k}\right))\right|}{)}^{2}. \end{array}$$ (35)

Now, using (35) in (31), we have

$$\begin{array}{c} \|d_{k}{\|}^{2}\leq 2(\frac{2\gamma \delta \beta_{k}}{\left|f(t_{k},h\left(t_{k}\right))\right|}{)}^{2}\|s_{k-1}{\|}^{2}+2{\left\|h\left(t_{k}\right)\right\|}^{2}. \end{array}$$

This can further be written as

$$\begin{array}{c} \|d_{k}{\|}^{2}\leq 2\phi_{k}^{2}\|s_{k-1}{\|}^{2}+2{\left\|h\left(t_{k}\right)\right\|}^{2}, \end{array}$$ (36)

where $\frac{2\gamma \delta \beta_{k}}{\left|f(t_{k},h\left(t_{k}\right))\right|}$.

Next, we estimate the modulus of *ϕ*_*k*_ by means of a so called property (*), this property was introduced by Gilbert and Nocedal [49] and recently extended in [1]. This property(*) indicates that *β*_*k*_ is small whenever *s*_*k*−1_ is small.

**Property** (*): Consider Algorithm 1 and assume that there exists $\bar{\delta }>0$ s.t

$$\begin{array}{c} \bar{\delta }\leq \left\|h\left(t_{k}\right)\right\|,\;\;\forall \;\;k\geq 1. \end{array}$$ (37)

Then we have property (*) if there exist *p* > 1 and λ > 0 s.t

$$\begin{array}{c} |\phi_{k}|\leq p, \end{array}$$ (38)

and

$$\begin{array}{c} \|s_{k-1}\left\|\leq \lambda \Rightarrow \right|\phi_{k}|\leq \frac{1}{2p}. \end{array}$$

We have by (28) and (38) that

$$\begin{array}{c} \frac{2\gamma \delta \beta_{k}}{\left|f(t_{k},h\left(t_{k}\right))\right|}\leq c_{1} \end{array}$$ (39)

holds with *c*_1_ = *p*/*γ*.

The result below indicates that, given some mild assumptions, a CG method fulfilling property (*) converges.

**Theorem 14** [1] *Given a CG Algorithm, assuming that Assumptions 9 and 11 are satisfied for all k, s.t (a)* *β*_*k*_ ≥ 0; *(b) d_k_ is a Q-DD of F at t*_*k*_; *(c) ℓ*_*k*_ *satisfies* (18); *(d) property* (*) *hold. Then*,

$$\begin{array}{c} \underset{k\to \infty }{\text{lim}\;\text{inf}}\;\left\|h\left(t_{k}\right)\right\|=0. \end{array}$$

**Remark 15** *It is evident that standard Wolfe* (17) *holds whenever strong Wolfe* (18) *is assumed. Thus, we assume only strong Wolfe condition for the subsequent results*.

**Theorem 16** *Let Assumptions 9 and 11 hold. Consider Algorithm 1, and let the sequence* {*t*_*k*_, *d*_*k*_} *be generated using any of the following*:

*(i)* $\beta_{k}=\beta_{k}^{PRP+}=\text{max}\left\{\beta_{k}^{PRP},0\right\}$ ;
*(ii)* $\beta_{k}=\beta_{k}^{HS+}=\text{max}\left\{\beta_{k}^{HS},0\right\}$ ;
*(iii)* $\beta_{k}=\beta_{k}^{HZ+}=\text{max}\left\{\beta_{k}^{HZ},0\right\}$ ;
*(iv)* $\beta_{k}=\beta_{k}^{DL+}=\text{max}\left\{\beta_{k}^{DL},0\right\}$ .

*If ℓ*_*k*_ *satisfies* (18), *then*,

$$\begin{array}{c} \underset{k\to \infty }{\text{lim}\;\text{inf}}\;\left\|h\left(t_{k}\right)\right\|=0. \end{array}$$ (40)

**Proof** We prove that condition (d) of Theorem 14 holds for all the cases (i)-(iv). To show this, we recall from [39] that, it is enough to show existence of a nonnegative constant *ϵ* s.t

$$\begin{array}{c} |\beta_{k}\left|\leq \epsilon \right\|s_{k-1}\|,\;\;\forall \;\;k\geq 1. \end{array}$$ (41)

Note that, by (37), Lemma 7(b), and (29), we get

$$\begin{array}{c} \frac{{\bar{\delta }}^{2}}{2}\leq -f\left(t_{k},h\left(t_{k}\right)\right)\leq \delta \gamma . \end{array}$$ (42)

(i) Beginning with $\beta_{k}=\beta_{k}^{PRP+},$ we have

$$\begin{array}{cc} |\phi_{k}| & =|\text{max}\{\frac{-f\left(t_{k},h\left(t_{k}\right)\right)+f\left(t_{k-1},h\left(t_{k}\right)\right)}{-f(t_{k-1},h\left(t_{k-1}\right))},0\}\frac{2\gamma \delta }{\left|f(t_{k},h\left(t_{k}\right))\right|}|\\ & \leq |(\frac{-f\left(t_{k},h\left(t_{k}\right)\right)+f\left(t_{k-1},h\left(t_{k}\right)\right)}{-f(t_{k-1},h\left(t_{k-1}\right))})\frac{2\gamma \delta }{\left|f(t_{k},h\left(t_{k}\right))\right|}|. \end{array}$$

Now, by Lemma 6(c)-(d) and (28), we get

$$\begin{array}{c} |-f\left(t_{k},h\left(t_{k}\right)\right)+f\left(t_{k-1},h\left(t_{k}\right)\right)\left|\leq L\right\|t_{k}-t_{k-1}\|\|h\left(t_{k}\right)\left\|\leq L\delta \right\|s_{k-1}\|, \end{array}$$ (43)

where ‖*s*_*k*−1_‖ = ‖*t*_*k*_ − *t*_*k*−1_‖.

Again, by (6) and (27), we estimate the following,

$$\begin{array}{c} |f\left(t_{k},s_{k-1}\right)\left|=\right|\left\langle JF\left(t_{k}\right)s_{k-1},\bar{q}\right\rangle |\leq ||JF\left(t_{k}\right)\left||\right\|s_{k-1}\left\|\leq \gamma \right\|s_{k-1}\|. \end{array}$$ (44)

Thus, by (43) and (42), we have

$$\begin{array}{c} |\phi_{k}|\leq (\frac{2L\delta \|s_{k-1}\|}{{\bar{\delta }}^{2}})\frac{4\delta \gamma }{{\bar{\delta }}^{2}}=\frac{8L\delta^{2}\gamma \left\|s_{k-1}\right\|}{{\bar{\delta }}^{4}}, \end{array}$$

where $\epsilon =\frac{8L\delta^{2}\gamma }{{\bar{\delta }}^{4}}.$ Therefore, we have property (*).

(ii) $\beta_{k}=\beta_{k}^{HS+},$ we have

$$\begin{array}{cc} |\phi_{k}| & =|\text{max}\{\frac{-f\left(t_{k},h\left(t_{k}\right)\right)+f\left(t_{k-1},h\left(t_{k}\right)\right)}{f\left(t_{k},d_{k}\right)-f\left(t_{k-1},d_{k-1}\right)},0\}\frac{2\gamma \delta }{\left|f(t_{k},h\left(t_{k}\right))\right|}|\\ & \leq |(\frac{-f\left(t_{k},h\left(t_{k}\right)\right)+f\left(t_{k-1},h\left(t_{k}\right)\right)}{f\left(t_{k},d_{k}\right)-f\left(t_{k-1},d_{k-1}\right)})\frac{2\gamma \delta }{\left|f(t_{k},h\left(t_{k}\right))\right|}|. \end{array}$$

Thus, by (43), (42) and (17), we have

$$\begin{array}{c} |\phi_{k}|\leq (\frac{2L\delta \|s_{k-1}\|}{\left(1-\sigma \right){\bar{\delta }}^{2}})\frac{4\delta \gamma }{{\bar{\delta }}^{2}}=\frac{8L\delta^{2}\gamma \left\|s_{k-1}\right\|}{\left(1-\sigma \right){\bar{\delta }}^{4}}, \end{array}$$

where $\epsilon =\frac{8L\delta^{2}\gamma }{\left(1-\sigma \right){\bar{\delta }}^{4}}.$ Therefore, we have property (*).

(iii) $\beta_{k}=\text{max}\left\{\beta_{k}^{HZ},0\right\}.$ Following similar arguments with previous estimate, we have

$$\begin{array}{c} |\phi_{k}|\leq \frac{1}{|\omega_{2}-\omega_{4}|}(|\omega_{1}\left|+\mu \right|\omega_{2}|\frac{|\omega_{1}+\omega_{3}|}{|\omega_{2}-\omega_{4}|})\frac{4\delta \gamma }{{\bar{\delta }}^{2}}. \end{array}$$

Thus, by (43), we have

$$\begin{array}{c} |\omega_{1}|=|-f\left(t_{k},h\left(t_{k}\right)\right)+f\left(t_{k-1},h\left(t_{k}\right)\right)\left|\leq L\delta \right\|s_{k-1}\| \end{array}$$ (45)

and by Lemma 6 (c)-(d)

$$\begin{array}{c} |\omega_{3}|=|f\left(t_{k},h\left(t_{k-1}\right)\right)-f\left(t_{k-1},h\left(t_{k-1}\right)\right)\left|\leq L\delta \right\|s_{k-1}\|. \end{array}$$ (46)

Observe that, by (17) and (24), we have

$$\begin{array}{c} f\left(t_{k},d_{k-1}\right)-f\left(t_{k-1},d_{k-1}\right)\geq -\left(1-\sigma \right)f\left(t_{k-1},d_{k-1}\right)\geq -\left(1-\sigma \right)f\left(t_{k-1},h\left(t_{k-1}\right)\right)>0. \end{array}$$

By Lemma 7 (b) and (37), we have

$$\begin{array}{c} \omega_{2}-\omega_{4}=f\left(t_{k},d_{k-1}\right)-f\left(t_{k-1},d_{k-1}\right)\geq \frac{\left(1-\sigma \right)\|h\left(t_{k-1}\right){)\|}^{2}}{2}\geq \frac{\left(1-\sigma \right){\bar{\delta }}^{2}}{2}>0. \end{array}$$ (47)

Again, by (17) and from (24), we have that *ω*_4_ < 0 for all *k*. Moreover,

$$\begin{array}{c} f\left(t_{k},d_{k-1}\right)\geq \sigma f\left(t_{k-1},d_{k-1}\right)=-\sigma (f\left(t_{k},d_{k-1}\right)-f\left(t_{k-1},d_{k-1}\right))+\sigma f\left(t_{k},d_{k-1}\right), \end{array}$$

using the definition of *ω*_2_ and *ω*_4_ and the fact that 0 < *σ* < 1, we get

$$\begin{array}{c} \omega_{2}\geq -\frac{\sigma }{1-\sigma }\left(\omega_{2}-\omega_{4}\right). \end{array}$$ (48)

Again, since *ω*_4_ < 0, for all *k*, we have that *ω*_2_ ≤ *ω*_2_ − *ω*_4_. Thus, by (47) and (48), we get

$$\begin{array}{c} \frac{|\omega_{2}|}{|\omega_{2}-\omega_{4}|}\leq \text{max}\{\frac{\sigma }{1-\sigma },1\}. \end{array}$$ (49)

Therefore, from (45), (46), (47), (48), and (49), we have

$$\begin{array}{c} |\phi_{k}|\leq \frac{8L\delta^{2}\gamma }{\left(1-\sigma \right){\bar{\delta }}^{4}}[1+2\mu \;\text{max}\{\frac{\sigma }{1-\sigma },1\}]\left\|s_{k-1}\right\|, \end{array}$$

where $\epsilon =\frac{8L\delta^{2}\gamma }{\left(1-\sigma \right){\bar{\delta }}^{4}}[1+2\mu \;\text{max}\{\frac{\sigma }{1-\sigma },1\}].$ Hence, we have property (*).

(iv) $\beta_{k}=\text{max}\left\{\beta_{k}^{DL},0\right\}.$ Then, we have

$$\begin{array}{c} |\phi_{k}|=|\text{max}\{\frac{-f\left(t_{k},h\left(t_{k}\right)\right)+f\left(t_{k-1},h\left(t_{k}\right)\right)}{f\left(t_{k},d_{k-1}\right)-f\left(t_{k-1},d_{k-1}\right)}-\alpha \frac{f(t_{k},s_{k-1})}{f\left(t_{k},d_{k-1}\right)-f\left(t_{k-1},d_{k-1}\right)},0\}\frac{4\delta \gamma }{{\bar{\delta }}^{2}}| \end{array}$$

$$\begin{array}{c} \;\;\leq (|\frac{-f\left(t_{k},h\left(t_{k}\right)\right)+f\left(t_{k-1},h\left(t_{k}\right)\right)}{f\left(t_{k},d_{k-1}\right)-f\left(t_{k-1},d_{k-1}\right)}|+\alpha |\frac{f(t_{k},s_{k-1})}{f\left(t_{k},d_{k-1}\right)-f\left(t_{k-1},d_{k-1}\right)}|)\frac{4\delta \gamma }{{\bar{\delta }}^{2}}. \end{array}$$

By (17) and (24), we have

$$\begin{array}{c} f\left(t_{k},d_{k-1}\right)-f\left(t_{k-1},d_{k-1}\right)\geq -\left(1-\sigma \right)f\left(t_{k-1},d_{k-1}\right)\geq -\left(1-\sigma \right)f\left(t_{k-1},h\left(t_{k-1}\right)\right)>0, \end{array}$$ (50)

for all *k* ≥ 1. Thus,

$$\begin{array}{c} |\phi_{k}|\leq (\frac{\left|-f\left(t_{k},h\left(t_{k}\right)\right)+f\left(t_{k-1},h\left(t_{k}\right)\right)\right|}{-\left(1-\sigma \right)f(t_{k-1},h\left(t_{k-1}\right))}+\alpha \frac{\left|f(t_{k},s_{k-1})\right|}{-\left(1-\sigma \right)f(t_{k-1},h\left(t_{k-1}\right))})\frac{4\delta \gamma }{{\bar{\delta }}^{2}}. \end{array}$$ (51)

Using (44) and (43) in (51), we get

$$\begin{array}{c} |\phi_{k}|\leq (\frac{L\delta \|s_{k-1}\|}{-\left(1-\sigma \right)f(t_{k-1},h\left(t_{k-1}\right))}+\frac{\alpha \gamma \|s_{k-1}\|}{-\left(1-\sigma \right)f(t_{k-1},h\left(t_{k-1}\right))})\frac{4\delta \gamma }{{\bar{\delta }}^{2}} \end{array}$$

$$\begin{array}{c} \;\;=(\frac{\left(L\delta +\alpha \gamma \right)\|s_{k-1}\|}{-\left(1-\sigma \right)f(t_{k-1},h\left(t_{k-1}\right))})\frac{4\delta \gamma }{{\bar{\delta }}^{2}}.\;\;\;\;\;\;\; \end{array}$$

We see by (42) that

$$\begin{array}{c} -\left(1-\sigma \right)f\left(t_{k-1},h\left(t_{k-1}\right)\right)\geq \frac{\delta^{2}\left(1-\sigma \right)}{2}. \end{array}$$

Therefore,

$$\begin{array}{c} |\phi_{k}|\leq \frac{8\delta \gamma \left(L\delta +\alpha \gamma \right)\|s_{k-1}\|}{\delta^{4}\left(1-\sigma \right)}. \end{array}$$

Hence, $\epsilon ≔\frac{8\delta \gamma (L\delta +\alpha \gamma )}{\delta^{4}\left(1-\sigma \right)}$ thereby completing the proof.

The lemma presented herein aligns with Lemma 6 as documented in [38]. It plays an important role in proving the convergence of the proposed CG methods.

**Lemma 17** *Suppose Assumptions 9 and 11 hold. Consider Algorithm 1, where ℓ_k_ is obtained by* (18). *If there exists* $\bar{\delta }>0$ *s.t*

$$\begin{array}{c} \|h\left(t_{k}\right)\|\geq \bar{\delta },\;\;\;for\;\;all\;\;k\geq 1 \end{array}$$ (52)

*and property* (*) *holds with β*_*k*_ ≥ 0. *Then, d*_*k*_ ≠ 0 *and*

$$\begin{array}{c} \sum_{k=0}^{\infty }\frac{1}{\|d_{k}{\|}^{2}}<\infty , \end{array}$$

$$\begin{array}{c} \;\;\sum_{k=0}^{\infty }{\left\|w_{k}-w_{k-1}\right\|}^{2}<\infty , \end{array}$$

*where* $w_{k}≔\frac{d_{k}}{\|d_{k}\|}$.

**Proof** Notice that *d*_*k*_ ≠ 0, otherwise (24) is not true. Thus, *w*_*k*_ is well-define. From the fact that *d*_*k*_ is *Q*-DD at *t*_*k*_ and *ℓ*_*k*_ fulfills (18), we have the Zoutendijk condition (30). Now, applying (52), Lemma 7 (b), and (7), we get

$$\begin{array}{c} \sum_{k=0}^{\infty }\frac{1}{\|d_{k}{\|}^{2}}\leq \frac{1}{{\bar{\delta }}^{4}}\sum_{k=0}^{\infty }\frac{\|h\left(t_{k}\right){\|}^{4}}{\|d_{k}{\|}^{2}}\leq \frac{4}{{\bar{\delta }}^{4}}\sum_{k=0}^{\infty }\frac{f^{2}\left(t_{k},h\left(t_{k}\right)\right)}{\|d_{k}{\|}^{2}}\leq \frac{4}{c^{2}{\bar{\delta }}^{4}}\sum_{k=0}^{\infty }\frac{f^{2}\left(t_{k},d_{k}\right)}{\|d_{k}{\|}^{2}}<\infty . \end{array}$$ (53)

Next, we define $u_{k}≔(h\left(t_{k}\right)-\frac{\beta_{k}}{f(t_{k},h\left(t_{k}\right))}f\left(t_{k},s_{k-1}\right)h\left(t_{k}\right))\frac{1}{\|d_{k}\|}$ and $\tau_{k}≔\ell_{k-1}\beta_{k}\frac{\|d_{k-1}\|}{\|d_{k}\|}.$ Then, (23) is rewritten as

$$\begin{array}{c} w_{k}=u_{k}+\tau_{k}w_{k-1}. \end{array}$$

Observe that

$$\begin{array}{c} \|w_{k}\left\|=\right\|w_{k-1}\|=1 \end{array}$$ (54)

and by applying (54), we have

$$\begin{array}{cc} \|\tau_{k}w_{k}-w_{k-1}{\|}^{2} & =\tau_{k}^{2}\|w_{k}\|-2\tau_{k}\left\langle w_{k},w_{k-1}\right\rangle +{\left\|w_{k-1}\right\|}^{2}\\ & =\tau_{k}^{2}\|w_{k-1}{\|}^{2}-2\tau_{k}\left\langle w_{k-1},w_{k}\right\rangle +{\left\|w_{k}\right\|}^{2}\\ & =\|\tau_{k}w_{k-1}-w_{k}{\|}^{2}. \end{array}$$

Now, we have

$$\begin{array}{c} \|u_{k}\left\|=\right\|\tau_{k}w_{k}-w_{k-1}\left\|=\right\|\tau_{k}w_{k-1}-w_{k}\|. \end{array}$$ (55)

It follows from *β*_*k*_ ≥ 0, (55) and the triangle inequality, that

$$\begin{array}{c} \begin{array}{cc} \|w_{k}-w_{k-1}\| & \leq \|\left(1+\tau_{k}\right)\left(w_{k}-w_{k-1}\right)\|\\ & \leq \|w_{k}-\tau_{k}w_{k-1}\left\|+\right\|\tau_{k}w_{k}-w_{k-1}\|\\ & =2\|u_{k}\|. \end{array} \end{array}$$ (56)

Since *s*_*k*−1_ = *t*_*k*_ − *t*_*k*−1_, then from (26), we have that $\|s_{k-1}\|\leq 2\bar{M}.$ Moreover, it follows from (28), (42) and (44) that

$$\begin{array}{c} \frac{\beta_{k}}{\left|f(t_{k},h\left(t_{k}\right))\right|}|f\left(t_{k},s_{k-1}\right)|\|h\left(t_{k}\right)\|\leq c_{1}\bar{M}. \end{array}$$

Also, from (56), we have

$$\begin{array}{c} \sum_{k=0}^{\infty }\|w_{k}-w_{k-1}{\|}^{2}\leq 4\sum_{k=0}^{\infty }{\left\|u_{k}\right\|}^{2} \end{array}$$

$$\begin{array}{c} \;\;\;\leq 4\sum_{k=0}^{\infty }(\|h\left(t_{k}\right)\|+\frac{\beta_{k}}{\left|f(t_{k},h\left(t_{k}\right))\right|}|f\left(t_{k},s_{k-1}\right)|\|h\left(t_{k}\right)\|{)}^{2}\frac{1}{\|d_{k}{\|}^{2}}. \end{array}$$

By (28) and (53), we get

$$\begin{array}{c} \sum_{k=0}^{\infty }{\left\|w_{k}-w_{k-1}\right\|}^{2}\leq 4{\left(\delta +\bar{M}c_{1}\right)}^{2}\sum_{k=0}^{\infty }\frac{1}{\|d_{k}{\|}^{2}}<\infty .\;\;\; \end{array}$$

This complete the proof.

Next, we present the main convergence theorem, considering *ϕ* instead of *β*_*k*_. The proof follows directly from [Theorem 2, p. 905, [38]], and thus, it is omitted.

**Theorem 18** *Given Algorithm 1 and assuming that Assumptions 9 and 11 hold. Then*

$$\begin{array}{c} \underset{k\to \infty }{\text{lim}\;\text{inf}}\;\left\|h\left(t_{k}\right)\right\|=0. \end{array}$$

## 4 Numerical results and discussions

In this section, we evaluate the performance of the proposed spectral-like Algorithm 1 by examining the following methods: PRP+, HS+, HZ, and DL+. We aim to gauge their efficiency and robustness in addressing benchmark test problems sourced from various MOO research articles. The algorithms were coded in Fortran 90. Subsequently, in the context of MOO, we define *e* as ${\left[1,\cdots ,1\right]}^{T}\in R^{m},$ *Q* as $R_{+}^{m},$ and *K* as $\left\{e_{1},e_{2},\cdots ,e_{m}\right\}\subset R^{m}$.

Below, we present a summary of the methods under consideration, including their initial parameter values. This encompasses both our proposed methods and those employed for comparison purposes:

- SPRP+: a spectral-like PRP+ method given by Algorithm 1 with *β*_*k*_ in (21);
- SHS+: a spectral-like HS+ method given by Algorithm 1 with *β*_*k*_ in (21);
- SHZ+: a spectral-like HZ method given by Algorithm 1 with *β*_*k*_ in (21) with *μ* = 1.0;
- SDL+: a spectral-like DL+ method given by Algorithm 1 with *β*_*k*_ in (21) and *α* = 0.1.

Our findings are compared with the following CG methods:

- HZ: a Hager-Zhang CG algorithm given in [38] with *μ* = 1.0,;
- SP: a spectral CG method (SCG) given in [43].

An essential part of the algorithms include computing the steepest descent direction, *h*(*t*). To achieve this, we utilize Algencan to solve problem (15); for more details, refer to [50]. In addition, the selection of the step size was performed using a LSE strategy that fulfills (18). The same LSE, employed for both HZ and SP, was used for all the proposed methods. Below are the initial parameters utilized in the LSE procedure for the implementation of the proposed methods:

$$\begin{array}{c} \rho =10^{-4},\;\sigma =0.1,\;c=0.4. \end{array}$$

On the other hand, we have by Lemma 7 that $t\in R^{n}$ is a stationary point if and only if *v*(*t*) = 0. Consequently, the experimentation was conducted by running all the implemented method up to the point of convergence, which is assumed to be $v\left(t\right)\geq -5\times eps^{\frac{1}{2}},$ or whenever, the maximum number of iterations, #*maxIt* = 5000 is exceeded. In this case, the *v*(*t*) is defined by (9) and the machine precision, *eps* ≈ 2.22 × 10^−16^.

Details of the test problems under consideration are provided in Table 1. The first column provides the names of the problem, for instance, “Lov1” aligning to the first problem introduced by A. Lovison in [51], and “SLCDT1” corresponding to the first problem given by Schütze, Lara, Coello, Dellnitz, and Talbi in [52]. All the remaining problems follow the same pattern with their corresponding references. The second column gives the corresponding references, while the third column is assigned for “*n*” the number of variables and the fourth column is assigned as “*m*” the objective functions of the problems. A box constraint was utilized for the starting points, defined as $\left\{t\in R^{n}\;|\;\bar{l}\leq t\leq \bar{u}\right\}$, where the lower bound is indicated in the fifth column and the upper bound is indicated in the last column.

**Table 1:** The considered test problems.

Problems	Refs.	n	m	${\bar{l}}^{T}$	${\bar{u}}^{T}$
JOS1	[54]	1000	2	(−10000, ⋯, −10000)	(10000, ⋯, 10000)
SLCDT1	[55]	2	2	(−5, −5)	(5, 5)
AP1	[33]	3	3	(−100, −100, −100)	(100, 100, 100)
AP2	[33]	2	2	(-100, -100)	(100, 100)
Lov1	[51]	2	2	(−100, −100)	(100, 100)
Lov3	[51]	2	2	(−100, −100)	(100, 100)
Lov4	[51]	2	2	(−100, −100)	(100, 100)
FF1	[54]	2	2	(−1, −1)	(1, 1)
FDS	[35]	3	3	(-2,-2, -2)	(2, 2, 2)
MMR1	[56]	2	2	(0, 0)	(1, 1)
MMR5	[56]	2	2	(−5, −5)	(5, 5)
MOP1	[54]	2	2	(−100000, −100000)	(100000, 100000)
MOP2	[54]	2	2	(−1, −1)	(1, 1)
MOP3	[54]	2	2	(−*π*, −*π*)	(*π*, *π*)
MOP5	[54]	2	3	(−1, −1)	(1, 1)
Far1	[54]	2	2	(−1, −1)	(1, 1)
SSFYY2	[54]	2	2	(−100, −100)	(100, 100)
SK1	[54]	2	2	(−100, −100)	(100, 100)
VU1	[54]	2	2	(−3, −3)	(3, 3)
Hil1	[57]	2	2	(0, 0)	(1, 1)
DD1	[54]	5	2	(−20, −20, −20, −20, −20)	(20, 20, 20, 20, 20))
KW2	[58]	2	2	(−3, −3)	(3, 3)
Toi4	[59]	4	2	(−100, −100, −100, −100)	(100, 100, 100, 100, 100)
Toi8	[59]	10	10	(−1, ⋯−1)	(1, ⋯ 1)
MGH26	[60]	4	4	(−1, −1, −1, −1)	(1,1,1,1)
MGH33	[60]	10	10	(−1, ⋯, −1)	(1, ⋯, 1)
PNR	[61]	2	2	(−1, −1)	(1, 1)
SLCDT2	[55]	10	3	(−100, ⋯, −100)	(100, ⋯, 100)
SLC2	[52]	1000	2	(−100, ⋯, −100)	(100, ⋯, 100)

In Table 2, the results of the proposed algorithms under the respective test problems are presented in comparison with HZ and SP CG methods. All the methods ran 100% successfully or reached a critical point. The table arrangements are: ‘Iter,’ ‘FunEva,’ and ‘GradEva.’ Thus, they denote the median number of iterations, functions, and gradient evaluations, respectively.

**Table 2:** Performance of the proposed spectral-like methods in comparison with HZ and SP.

Problem	SPRP+			SHS+			SHZ+		
	Iter	FunEva	GradEva	Iter	FunEva	GradEva	Iter	FunEva	GradEva
**JOS1**	1	2	4	1	2	4	1	2	4
**SLCDT1**	3	22	21	3	22	21	3	22	21
**AP1**	11	104	85	11	104	85	8.5	79.5	68
**AP2**	1	2	4	1	2	4	1	2	4
**Lov1**	3	6	8	3	6	8	3	6	8
**Lov3**	3	9	11	3	9	11	3	9	11
**Lov4**	2	6	8	2	6	8	2	6	8
**FF1**	12	77.5	66	12	77.5	66.5	12	75	65
**FDS**	13.5	119.5	99.5	13.5	119.5	99.5	14	119.5	99.5
**MMR1**	8	56	42	7.5	53.5	42	8	56	42
**MMR5**	51	260.5	243.5	62.5	371.5	346.5	56	293	270
**MOP1**	1	2	4	1	2	4	1	2	4
**MOP2**	7	52.5	43	7	55.5	51.5	7	51	42.5
**MOP3**	8.5	33.5	33.5	8	37	34.5	8	33.5	33.5
**MOP5**	2	18	19	2	19	20	2	18	19
**Far1**	27	152	131	29.5	157.5	137.5	28.5	167.5	138.5
**SSFYY2**	1	9	10	1	9	10	1	9	10
**SK1**	2	18	18	2	18	18	2	18	18
**VU1**	760	2287	2289	760	2287	2289	760	2287	2289
**Hil1**	10	65	55	10	72.5	60.5	10	65	55
**DD1**	29.5	101	103	30	102.5	104.5	30	104	106
**KW2**	11.5	76.5	66	12	85.5	73	11	78	69
**Toi4**	4	22	21	5	28	26	4	22	21
**Toi8**	1	26	33	1	26	33	1	26	33
**MGH26**	5	63.5	56	4	54.5	53	4.5	63.5	55.5
**MGH33**	1	22	31	1	22	31	1	22	31
**PNR**	1	3	5	1	3	5	1	3	5
**SLCDT2**	20	183	161.5	18	172	153.5	21	186	162
**SLC2**	23.5	161	139	28.5	188.5	172	27	188.5	161

In a VOP setting, the primary objective is to approximate the Pareto frontier of the given problem. To achieve this, we employed a methodology where each implemented method underwent 200 runs for each problem, and Iter, FunEva, and GradEva were recorded for each run. The methods began with initialization using uniformly distributed random points within the problem’s specified bounds, as detailed in Table 1. The comparison metrics employed here include the Iter, FunEva, and GradEva.

In order to guarantee an equitable and significant comparison of algorithms, we employed the well-established performance profile as documented in [53]. The profile visually represents algorithm performance, which compares algorithmic performance across various metrics, offering a comprehensive assessment of efficiency and robustness. This tool enables us to concisely summarize the experimental data showcased in Tables 2 and 3. Furthermore, the performance profile offers insights into the effectiveness of the methods proposed in this study in comparison to the HZ and SP CG methods.

**Table 3:** Continuation of Table 2.

SDL+			HZ			SP		
Iter	FunEva	GradEva	Iter	FunEva	GradEva	Iter	FunEva	GradEva
1	2	4	1	2	4	1	2	4
3	22	21	3	22	21	3	21	21
5	59	54.5	11	104	85	11	104	86
1	2	4	1	2	4	1	2	4
3	6	8	4.5	9	11	2	4	6
3	9	11	9	35	33	3	9	11
2	6	8	4.5	18.5	19.5	2	6	8
12	78.5	67	8	55	50	12.5	78.5	68
14	120.5	101	8	86	77.5	14	119.5	99.5
7.5	53.5	42	7	54	42	7	53	42
60.5	368	338.5	82	419	395	112	883.5	977
1	2	4	1	2	4	1	2	4
7	52.5	43	7	50	44	6	44	39
8	38.5	34	9	42	44	9	40.5	37.5
2	19	20	2	18	19	3	23	24
29.5	157.5	137.5	27	166.5	146	45	345.5	340.5
1	9	10	2	10	11	2	10	11
2	18	18	2	18	19	2	24	24
760	2287	2289	762	2293	2295	762	2293	2295
10	68.5	58	10	65	57.5	10	68.5	56.5
30	102.5	104.5	32	112	114	31.5	112	114
12	89	73.5	14	101.5	86.5	14	110.5	99.5
5	28	26	4	24	22	4	24	21
1	26	33	1	27	33	1	26	33
4	58	54	5	64	59	4	61	56.5
1	22	31	1	22	33	1	22	33
1	3	5	1	3	5	1	3	5
19	182	158	21.5	236.5	210.5	16	172	160
31	210	181.5	36	273	239.5	29.5	195	182

**Fig 1:**
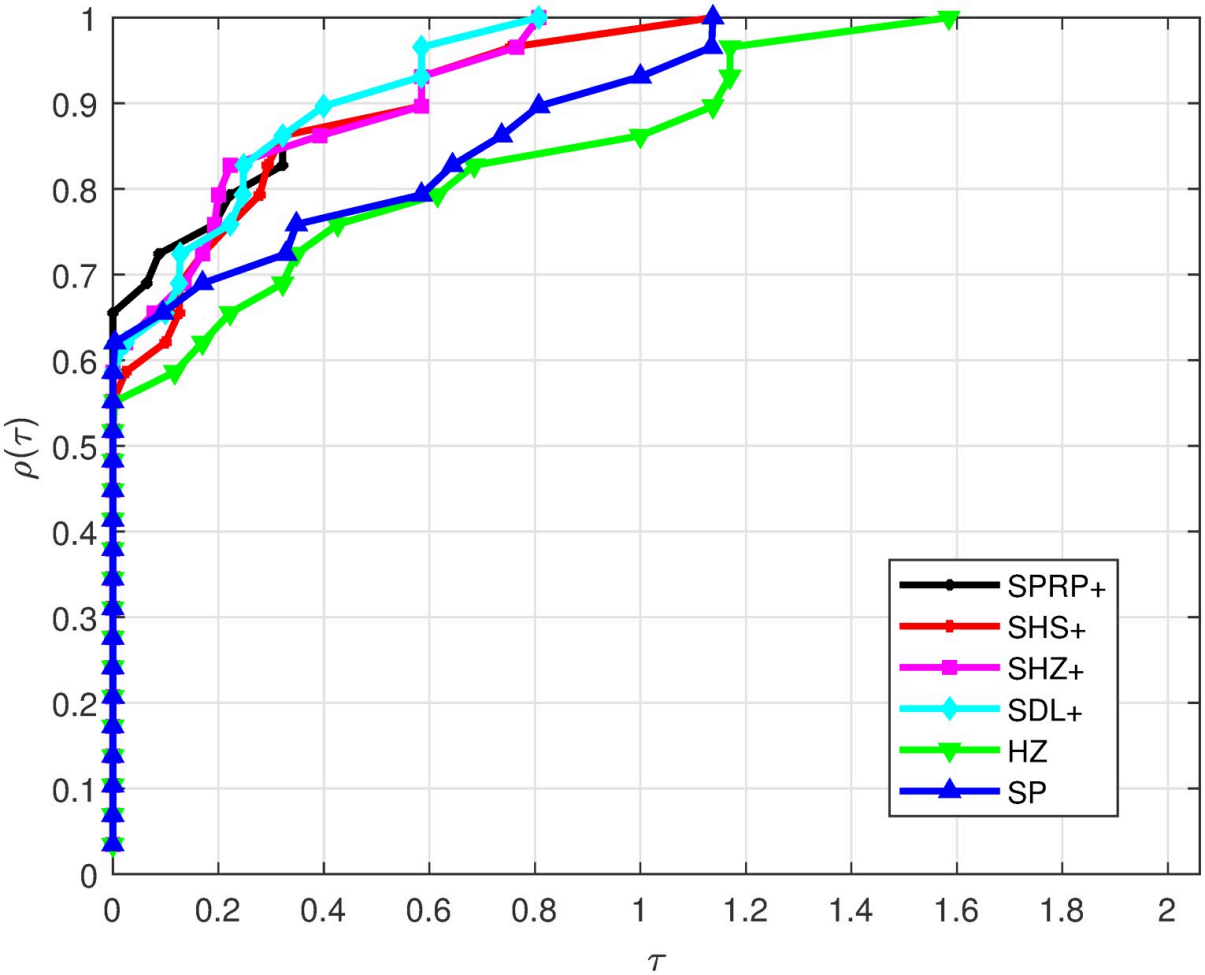
Performance on Iter.

**Fig 2:**
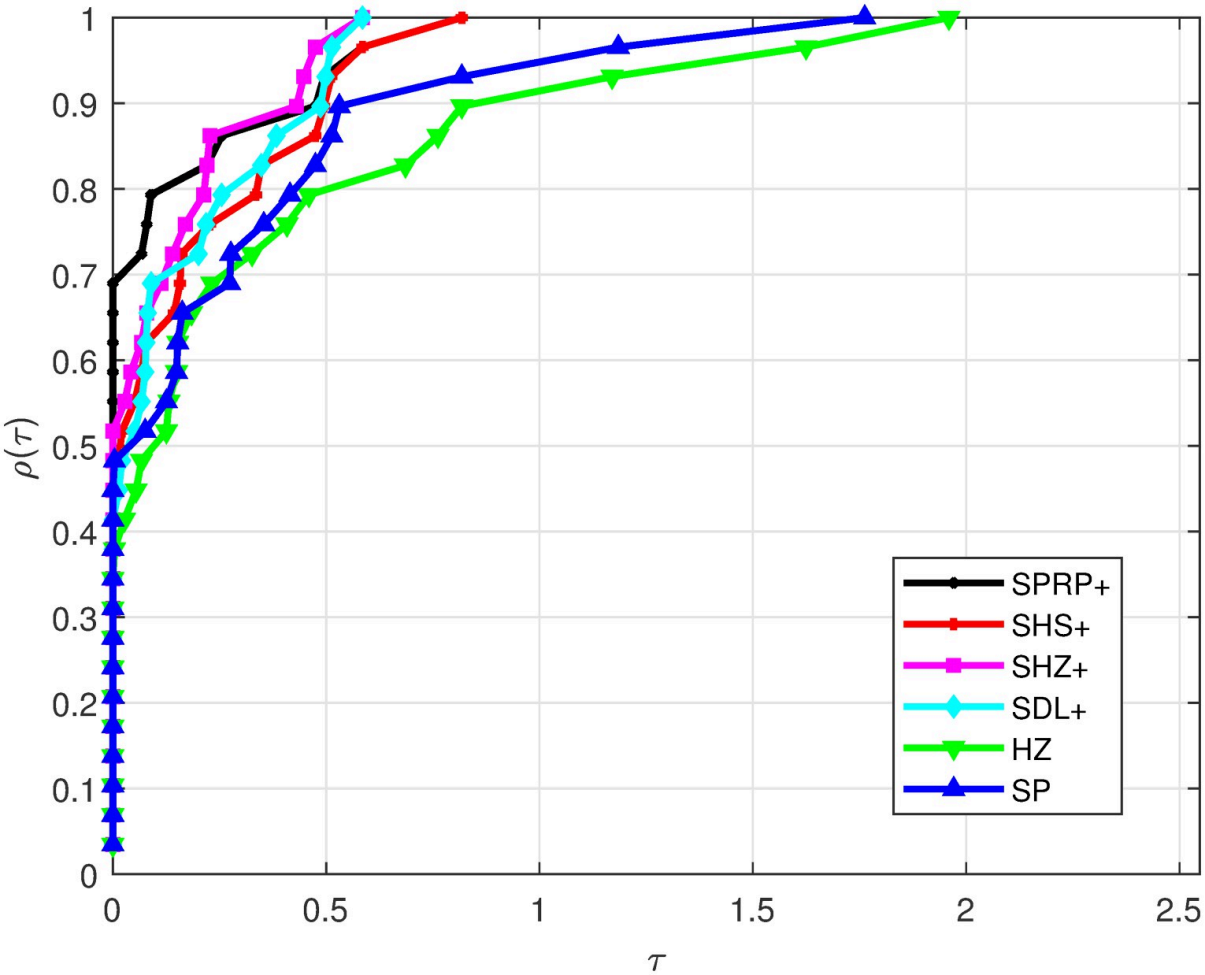
Performance on FunEva.

**Fig 3:**
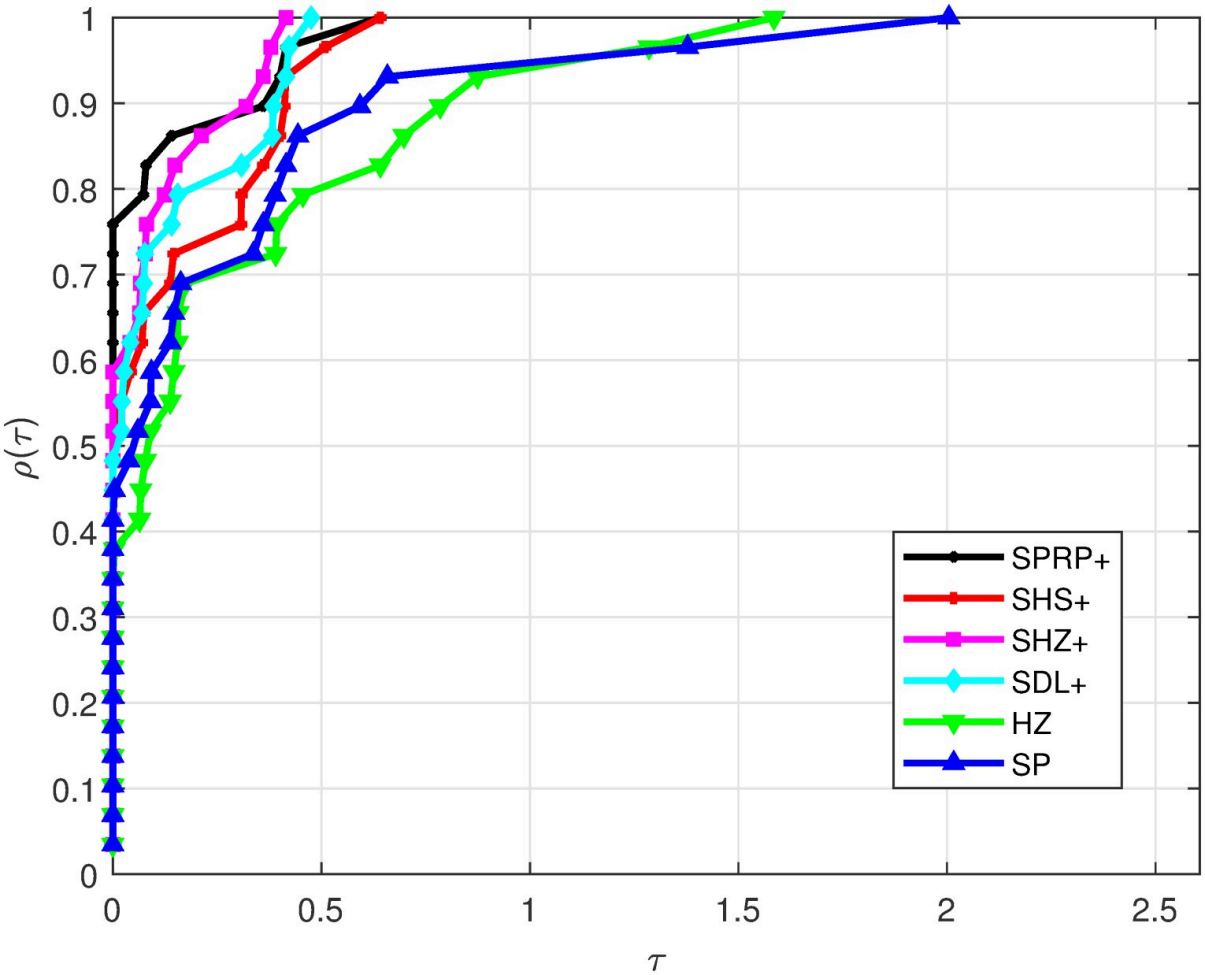
Performance on GradEva.

Based on the considered test problems, the performance profile for Iter is displayed in Fig 1. It is observed that the SPRP+, SHZ+, SDL+, and SHS+ methods exhibit the best performance according to their appearance, outperforming the other compared methods. On the other hand, the HZ and SP methods are the least-performing. Additionally, Fig 2 illustrates FunEva, showing that the SPRP+, SHZ+, SDL+, and SHS+ methods required fewer function evaluations compared to the HZ and SP methods. Finally, Fig 3 presents GradEva, where the SPRP+, SHZ+, SDL+, and SHS+ methods evaluated fewer gradients than the HZ and SP methods. This observation aligns with the fact that our methods satisfy the SDC independently of any LSE.

## 5 Concluding remarks

We introduced new spectral-like CG methods that achieve sufficient descent property independently of any LSE and for arbitrary nonnegative CG parameters. Four well-known conjugate parameters, PRP+, HS+, HZ+, and DL+ are considered and thus are referred to as SPRP+, SHS+, SHZ+, and SDL+, respectively. We established the convergence of the proposed methods using Wolfe LSE. Our algorithms achieved this without regular restart and assumption of convexity regarding the objective functions. The sequences generated by our algorithm identify points that satisfy the first-order necessary condition for Pareto optimality. We conducted computational experiments which show the implementation and efficiency of the methods with a promising performance. The proposed spectral-like methods, SPRP+, SHZ+, SDL+, and SHS+, exhibited the best performance according to their appearance, outperforming HZ, and SP methods in all the considered metrics, the number of iterations, function, and gradient evaluations.

A challenging task to consider in the future is the three-term CG method, which is of special interest in yielding sufficient descent of the search direction. This work may be challenging, considering that *f* as defined in (6) is only sublinear with respect to the second variable. However, the work herein provides insight into the three-term method.
